# Neutrophil and macrophage crosstalk might be a potential target for liver regeneration

**DOI:** 10.1002/2211-5463.13803

**Published:** 2024-05-06

**Authors:** Yiyuan Chen, Yijie Yang, Jinjiao Lu, Huan Chen, Zhixiong Shi, Xiaodong Wang, Nan Xu, Xiao Xu, Shuai Wang

**Affiliations:** ^1^ The Fourth School of Clinical Medicine Zhejiang Chinese Medical University, Affiliated Hangzhou First People's Hospital Hangzhou China; ^2^ Zhejiang University School of Medicine Hangzhou China; ^3^ Key Laboratory of Integrated Oncology and Intelligent Medicine of Zhejiang Province Hangzhou China; ^4^ Institute of Organ Transplantation Zhejiang University Hangzhou China

**Keywords:** crosstalk, immune cells, liver regeneration, macrophages, neutrophils, partial hepatectomy

## Abstract

The regenerative capability of the liver is remarkable, but further research is required to understand the role that neutrophils play in this process. In the present study, we reanalyzed single‐cell RNA sequencing data from a mouse partial hepatectomy (PH) model to track the transcriptional changes in hepatocytes and non‐parenchymal cells. Notably, we unraveled the regenerative capacity of hepatocytes at diverse temporal points after PH, unveiling the contributions of three distinct zones in the liver regeneration process. In addition, we observed that the depletion of neutrophils reduced the survival and liver volume after PH, confirming the important role of neutrophils in liver regeneration. CellChat analysis revealed an intricate crosstalk between neutrophils and macrophages promoting liver regeneration and, using weighted gene correlation network analysis, we identified the most significant genetic module associated with liver regeneration. Our study found that hepatocytes in the periportal zone of the liver are more active than in other zones, suggesting that the crosstalk between neutrophils and macrophages might be a potential target for liver regeneration treatment.

## Graphical abstract

Neutrophils interact with macrophages increase the secretion of interleukin (IL)‐6 and hepatocyte growth factor (HGF). This interaction between macrophages and hepatocytes, in turn, promotes liver regeneration. Neutrophils also stimulate the differentiation of monocytes into macrophages, which accelerates liver proliferation. Neutrophil depletion results in a decreased interaction with monocytes and macrophages, which decreases liver regeneration.

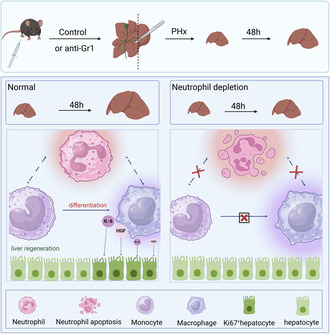

**Abbreviations**
ALT: alanine aminotransferase
AST: aspartate aminotransferase
Ccl: C‐C motif chemokine ligand
Cxcl: C‐X‐C motif chemokine ligand
HCC: hepatocellular carcinoma
HFD: high fatty diet
IL: interleukin
KC: Kupffer cell
KEGG: Kyoto Encyclopedia of Genes and Genomes
Lcn2: lipocalin‐2
Mt: metallothionein
NPCs: non‐parenchymal cells
Orm: orosomucoid
PH: partial hepatectomy
qRT‐PCR: quantitative real‐time PCR
scRNA‐seq: single‐cell RNA sequencing
t‐SNE: t‐distributed stochastic neighbor embedding
WGCNA: weighted gene correlation network analysis

Despite the array of available treatment modalities and potential combination therapies, the prognosis for hepatocellular carcinoma (HCC) remains dismal, with a 5‐year survival rate of approximately 18% [1, 2, 3]. Liver transplantation and surgical resection are the most effective approaches for treating HCC [4]. The extraordinary regenerative prowess of the adult liver allows for recuperation following radical resection or transplantation [5]. However, because most patients with HCC present with underlying liver disease [6], liver resection or transplantation necessitates a concerted effort to preserve as much parenchymal tissue as possible, thus mitigating the risk of liver failure [7]. Therefore, exploring the mechanism underlying liver regeneration is of profound significance.

The immune system plays an indispensable role in the orchestration of liver regeneration. A multifaceted interplay of immune components, including immune cells, cytokines, complements, immunoglobulins and cell adhesion molecules, substantially contributes to liver regeneration [8, 9]. Moreover, the immune microenvironment is pivotal in liver inflammatory diseases and immunocompromised liver conditions [10]. Neutrophils play a pivotal role in liver diseases, including cancer, making them attractive targets for the treatment of these diseases [11]. In the present study, we investigated the role of neutrophils in liver regeneration. Neutrophils possess sophisticated machinery for essential functions in immunity and inflammation. Toll‐like receptor 5‐mediated signaling accelerates neutrophil and monocyte recruitment during the priming phase, expediting liver regeneration [12]. Furthermore, in the progression phase, neutrophils actively phagocytose apoptotic extracellular vesicles, leading to the secretion of hepatocyte growth factor and fibroblast growth factor‐2, further promoting liver regeneration [13]. Notably, after partial hepatectomy (PH) or associated liver partition and portal vein ligation for staged hepatectomy, neutrophils and macrophages undergo a phenotypic transition from pro‐inflammatory to pro‐regenerative states as they diligently clear cell debris [14, 15].

In the present study, we conducted a meticulous reanalysis of single‐cell RNA sequencing (scRNA‐seq) data derived from a mouse PH model, employing cutting‐edge methodologies, including weighted gene correlation network analysis (WGCNA), and pseudotime analysis to unravel the regenerative capacity of hepatocytes at diverse temporal points and the contributions of three distinct zones in the liver regeneration process. CellChat analysis (Suoqin Jin, CA, USA, https://github.com/sqjin/CellChat) aimed to trace the intricate cellular transitions and heterogeneities within immune cells during the initiation and progression peak phases of liver regeneration.

## Materials and methods

### scRNA‐seq data processing

The scRNA‐seq data of the PH samples were processed using r, version 4.2.1 (R Foundation, Vienna, Austria) and the Seurat package [16]. The harmony package (Ilya Korsunsky, MA, USA, https://github.com/immunogenomics/harmony) was used to remove batch effects. After eliminating genes expressed in < 10 cells and cells expressing < 200 genes, cell samples with a mitochondrial gene proportion < 15% were included in the subsequent analysis and logarithmically normalized. We conducted principal component analysis on the scaled data and implemented a graph‐based clustering approach. We harnessed t‐distributed stochastic neighbor embedding (t‐SNE) to visualize and explore the datasets. monocle, version 2.0 (cole‐trapnell‐lab.github.io) was used for pseudotime analysis in accordance with the online documentation [17].

### Analysis of differential gene enrichment of clusters

The marker genes were determined using the ClusterProfilerand org.Mu.eg.db packages (https://bioconductor.org/packages/release/bioc/html/ClusterProfiler.html; https://www.bioconductor.org/packages/release/data/annotation/html/org.Hs.eg.db.html). Gene Ontology (http://geneontology.org) and Kyoto Encyclopedia of Genes and Genomes (KEGG) (https://www.genome.jp/kegg) enrichment analyses were performed, using *q* < 0.05 to determine statistically significant enrichment. We used the scMetabolism package to analyze the enrichment of metabolism pathways [16].

### Identification of modules of correlated genes and link modules with traits

WGCNA can be used to find modules of correlated genes and identify disease‐related biomarkers. We used the WGCNA package [18] to identify the regenerative‐associated marker genes. The analysis involved generating similarity, adjacency and topological overlap matrices through Pearson correlation analysis. A soft threshold power of 18 was chosen to define the adjacency matrix based on the criterion of approximate scale‐free topology, and 0.8 was used as the scale independence. Subsequently, a tree diagram was plotted and hierarchical clustering grouped the modules related to clinical traits. The final correlation module (*P* < 0.05) was the basis for the subsequent analyses.

### Cell–cell communication analysis

CellChat was used to analyze cell‐to‐cell communication in a single dataset. We inspected the cell type‐specific RNA expression of various ligands in the liver secretome and their corresponding receptors, mapping numerous unique clusters of ligand‐receptor pairs with cell type–specific expression patterns. We also used CellChat to analyze and visualize the data [19].

### Animal experiments

Six‐ to eight week‐old male C57BL/6 mice were bought from the Model Animal Research Center of Nanjing University (Nanjing, China) and randomly assigned to each group (*n* = 10). All animal experiments were performed up to standard followed by the Ethics Committee of the Institutional Animal Care and Use Committee (IACUC) (approval number: ZJCLA‐IACUC‐20050066). We used a 70% PH model as first reported by Higgins and Anderson and modified by Boyce [20, 21]. PH is a widely used model of liver regeneration that closely resembles clinical liver recession, and its structure is similar to that of clinical liver recession. During the surgical procedure, when mice were under anesthesia (inhalation anesthesia with 5% isoflurane during induction and 3% isoflurane for maintenance), the right median (30%), left median (10%) and left lateral (30%) lobes of the liver were removed. Each of these lobes was tied off at its base using a preformed silk loop, which was manipulated around the lobes before being secured. We killed the mice 48 h after PH because the peak of the mice's regeneration phase occurred between 36 and 48 h [20, 21]. The liver‐to‐body weight ratio was calculated using the weight of lobes to delineate the increasing rate of liver volume (liver‐to‐body weight ratio = liver weight/body weight, g·g^−1^).

InVivoMAb anti‐mouse LY6G (BP0075‐1; Bio X Cell, Lebanon, NH, USA), InVivoMAb anti‐mouse Ly6G/Ly6C (Gr‐1) (BE0075; Bio X Cell) and anti‐rat IgG2a (BE0122; Bio X Cell) were used to deplete neutrophils, as previously reported [22]. Rat IgG2a isotype was used as a control (BP0089; Bio X Cell) used as control (control: 50 μg·day^−1^ 7 days prior and once after PH; anti‐Gr1: 50 μg·day^−1^ 7 days prior and once after PH, combo: 50 μg·day^−1^ anti‐Ly6G + 50 μg·day^−1^ anti‐rat IgG2a 7 days prior and once after PH).

### Cell culture

Monocytes were harvested from the bone marrow of C57BL/6 mice, then purified using gravity centrifugation (520 ***g*** for 5 min) and seeded in an incomplete medium. The unattached cells were removed and the adherent cells were added to a complete 1640 medium after 6 h of incubation under 5% CO_2_ at 37 °C. Mouse primary neutrophils were isolated following a protocol reported for the isolation of mouse neutrophils [23]. Neutrophils were purified using Histopaque1077 (Sigma, St Louis, MO, USA) and Histopaque1119 (Sigma) under gravity centrifugation (800 ***g*** for 30 min). Neutrophils were incubated with a complete 1640 medium (5% CO_2_ at 37 °C). Complete 1640 medium comprised 10% fetal bovine serum (Gibco, Waltham, MA, USA), 100 IU·mL^−1^ penicillin and 100 μg·mL^−1^ streptomycin.

### Flow cytometry

Single cells were isolated and digested using a modified liver collagenase perfusion method from the livers of the control and PH mice [24]. We used a 70‐mm filter (BD Biosciences, Franklin Lakes, NJ, USA) to perfuse livers. Total immune cells were purified using medium‐speed gravity centrifugation (520 ***g*** for 5 min × 3). Live cells were stained using Fixable Viability Stain 780 (dilution 1 : 1000; 565388; BD Biosciences) and performed in FACS buffer (4 °C for 30 min). Primary antibodies against CD45 (dilution 1 : 100; 75‐0451‐U100; TONBO, Tokyo, Japan), CD11b (dilution 1 : 100; 85‐0112‐U100; TONBO), Ly6G (dilution 1 : 100; 127633; BioLegend, San Diego, CA, USA), F4/80 (dilution 1 : 100; 123137; BioLegend), LY6C (dilution 1 : 100; 20‐5932‐U100; TONBO) and CLEC4F (dilution 1 : 100; 156804; BioLegend) were carried out in FACS buffer (4 °C for 60 min). Cells were sorted using a BD Aria III, and data were analyzed using flowjo, version 10 (https://www.flowjo.com).

### Quantitative real‐time PCR

We utilized the Trizol Reagent (Invitrogen, Waltham, MA, USA) to extract the total RNA and the PrimeScript RT Master Mix kit (Takara, Shiga, Japan) to reverse‐transcribe into cDNA. Quantitative real‐time PCR (qRT‐PCR) was performed using the Q6 real‐time PCR system (Applied Biosystems, Waltham, MA USA) with the SYBR Green Master Mix (Takara). Target genes were normalized to GAPDH expression levels. Each group had three biological replicates, and each sample had three technical replicates. The primers used for qRT‐PCR were: Gapdh (forward: 5′‐AGGTCGGTGTGAACGGATTTG‐3′, reverse: 5′‐GGGGTCGTTGATGGCAACA‐3′), Ccl2 (forward: 5′‐TTAAAAACCTGGATCGGAACCAA‐3′, reverse: 5′‐GCATTAGCTTCAGATTTACGGGT‐3′), Ccl3 (forward: 5′‐TTCTCTGTACCATGACACTCTGC‐3′, reverse: 5′‐CGTGGAATCTTCCGGCTGTAG‐3′), Ccl5 (forward: 5′‐GCTGCTTTGCCTACCTCTCC‐3′, reverse: 5′‐TCGAGTGACAAACACGACTGC‐3′), Cxcl1 (forward: 5′‐CTGGGATTCACCTCAAGAACATC‐3′, reverse: 5′‐CAGGGTCAAGGCAAGCCTC‐3′), Sox9 (forward: 5′‐GAGCCGGATCTGAAGAGGGA‐3′, reverse: 5′‐GCTTGACGTGTGGCTTGTTC‐3′), Ki67 (forward: 5′‐GAGGAGAAACGCCAACCAAGAG‐3′, reverse: 5′‐TTTGTCCTCGGTGGCGTTATCC‐3′), hepatocyte growth factor (forward: 5′‐ATGTGGGGGACCAAACTTCTG‐3′, reverse: 5′‐GGATGGCGACATGAAGCAG‐3′), Pcna (forward: 5′‐TTTGAGGCACGCCTGATCC‐3′, reverse: 5′‐GGAGACGTGAGACGAGTCCAT‐3′).

### Immunofluorescence staining and immunohistochemical staining

The liver sections (4 μm thick) were embedded, sliced, dried, dewaxed and rehydrated for the experiments. Next, they were quenched with 3% H_2_O_2_ (if needed), followed by heat‐induced epitope retrieval in citrate buffer (10 mm, pH 6, 95 °C for 20 min). We used 5% BSA (A7906; Sigma) to block the non‐specific antigens. Anti‐Ly6G antibody (dilution 1 : 200, ab238132; Abcam, Cambridge, MA, USA), anti‐Ki67 antibody (dilution 1 : 500, ab15580; Abcam) and anti‐Pcna antibody (dilution 1 : 500, ab18197; Abcam) were incubated overnight at 4 °C. Horseradish peroxidase‐conjugated goat‐anti‐rabbit IgG (dilution 1 : 100, PR30011; Proteintech, Rosemont, IL, USA) and CoraLite488‐conjugated goat‐anti‐rabbit IgG or rhodamine‐conjugated goat‐anti‐mouse IgG (dilution 1 : 100, SA00013‐2, SA00007‐2; Proteintech) were used as secondary antibody at 4 °C for 1 h. Immunofluorescence used 4′,6‐diamidino‐2‐phenylindole for nucleus staining (Abcam), whereas immunohistochemical staining of the nucleus was performed using hematoxylin. Slides were mounted and visualized using a microscope (Olympus, Tokyo, Japan).

### Serum analysis

Mouse serum alanine aminotransferase (ALT) and aspartate aminotransferase (AST) were measured using an ALT and AST Assay kit (E‐BC‐K235‐M, E‐BC‐K236‐M; Elabscience, Wuhan, China) in accordance with the manufacturer's instructions.

### Statistical analysis

No pre‐processing of the data was carried out. For all experiments, mice were randomly assigned to groups of 10 mice. Only dead mice were excluded from the experiments. The immunohistochemical and immunofluorescence staining experiments were conducted in a double‐blinded manner, ensuring the unbiasedness and reliability of the results. We used image j, version 1.53t (NIH, Bethesda, MD, USA) to quantify each liver sample, and all liver sections were repeated at least three times. Data were analyzed using prism, version 8.0 (GraphPad Software Inc., San Diego, CA, USA) and the results are presented as the mean ± SD. All the immunofluorescence, immunohistochemistry, flow cytometry and qPCR data were repeated at least thrice to quantify liver tissues. We used the Shapiro–Wilk test [25] to check the normal distribution of characteristics (*P* > 0.05). An unpaired two‐tailed Student's *t*‐test was used to determine statistical significance between two groups and multiple group data were analyzed using one‐way analysis of variance when the data conformed to a normal distribution. A chi‐square test was used to analyze abnormal distributions. *P* < 0.05 was considered statistically significant.

## Results

### Hepatocyte subsets analysis during liver regeneration

**Fig. 1:**
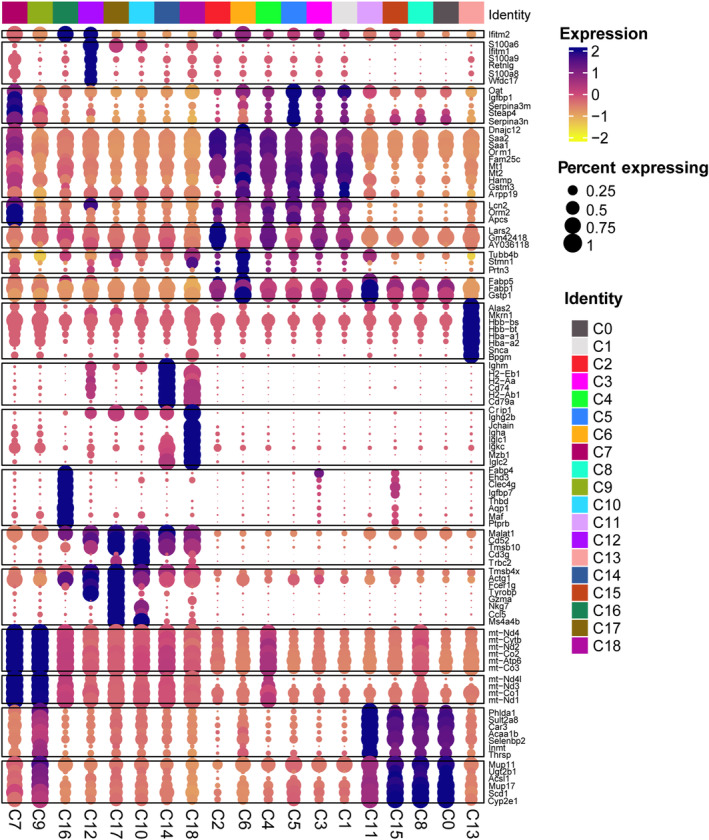
The definition of 19 clusters. Clusters 0–18 were identified using enrichment genes. The intensities and directions of the correlations are indicated on the right side of the diagram. The color and cluster names are shown on the right.

**Fig. 2:**
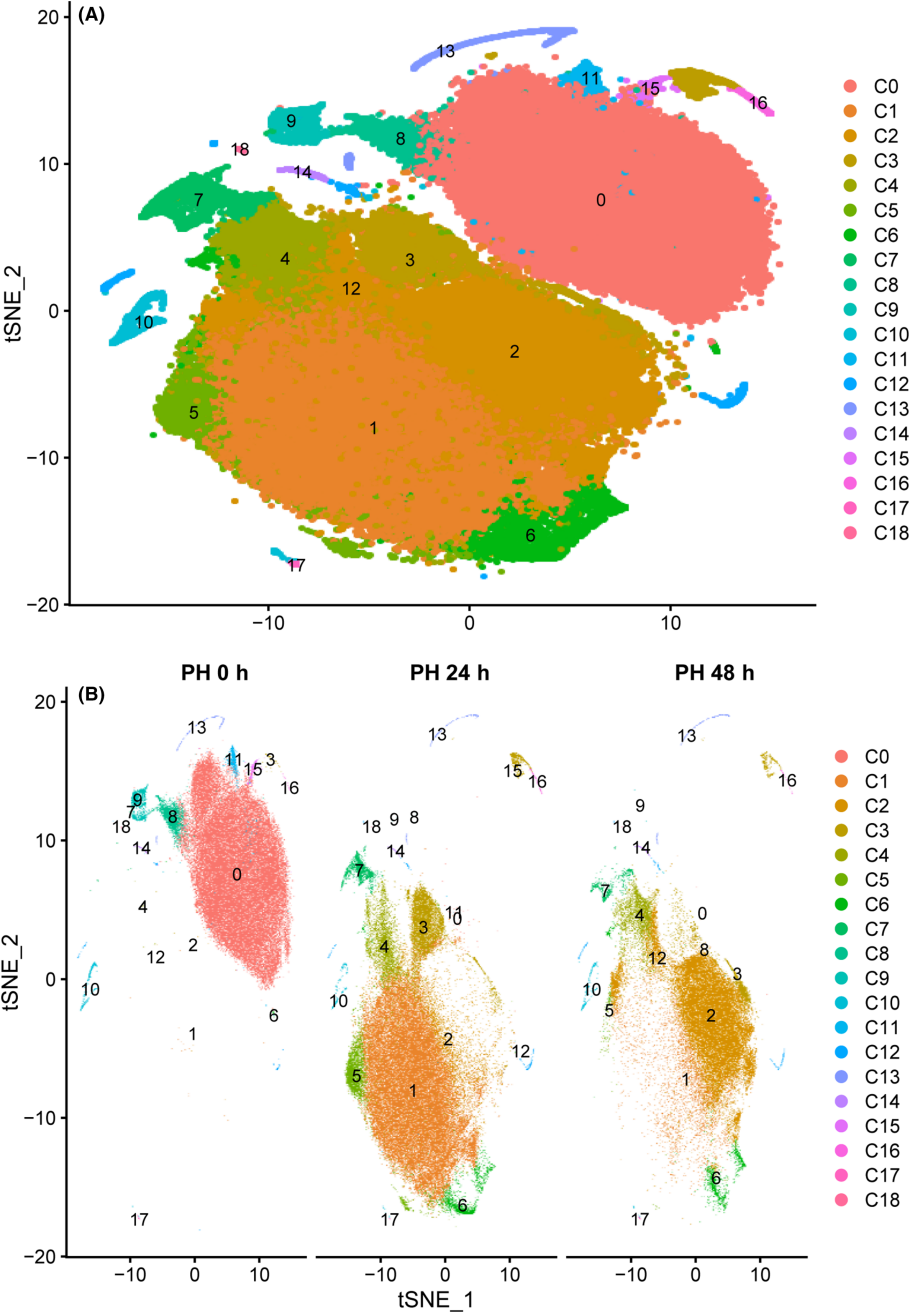
Cell type composition of a regenerating liver and dynamically changed hepatocytes during liver regeneration. (A) Cells were classified into 18 subsets using the t‐SNE algorithm. (B) Cells were separated at 0, 24 and 48 h after PH, using the t‐SNE algorithm.

**Fig. 3:**
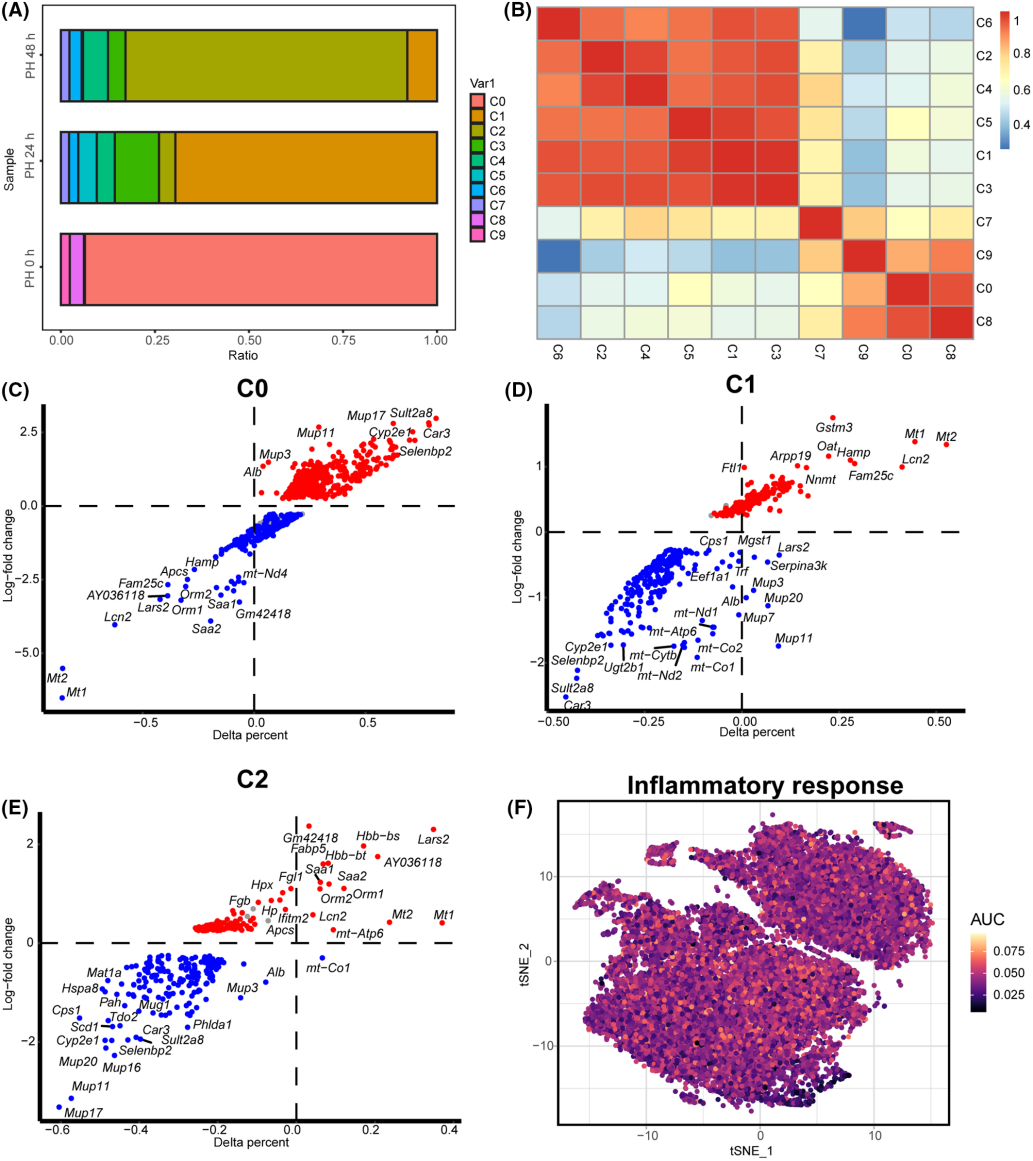
The regenerative capacity of hepatocytes after PH. (A) Proportions of the cells for clusters 0–9 at 0, 24 and 48 h after PH. (B) Heat map of relative expression of the top genes enriched in hepatocytes. The intensity and direction of the correlations are indicated on the right side of the heatmap. (C–E) Expression patterns of critical genes in cluster 1 and cluster 2 are shown as volcano plots. (F) Distribution of the inflammatory response metabolic pathways in clusters 0–9, using the t‐SNE algorithm.

We first analyzed scRNA‐seq from adult mouse livers 0, 24 and 48 h after PH. In total, we recovered 199 229 single cells that passed quality control. We identified 19 clusters in liver samples. Clusters 0–9 were identified as hepatocytes based on the presence of parenchymal cell marker genes (Alb, Cyp2e1 and Oat), and clusters 10–18 were non‐parenchymal cells (NPCs) (for definition details, see below) (Fig. 1). During PH, the main cell subsets were clusters 0, 1 and 2 at 0, 24 and 48 h, respectively (Figs 2A,B and 3A). Moreover, cell correlation analysis showed clusters 0, 7, 8 and 9 were different from clusters 1, 2, 3, 4, 5 and 6. The above comment shows that clusters 0, 7, 8 and 9 were quiescent cells, whereas clusters 1–6 were proliferative cells (Fig. 3B). Cluster 0 (Sult2a8, Cyp2e1 and Alb) is the main hepatocyte subset that exists in normal liver tissue (Fig. 3C); mup11, mup3 and mup17 were highly expressed in cluster 0 and expressed at low levels in clusters 1 and 2, indicating that synthetic liver function may be damaged at 24 and 48 h after PH [26]. Interestingly, cluster 1 (Mt1/2, Gstm3, Oat) and cluster 2 (Mt1/2, Saa1/2, Ifith2) were the major components at 24 and 48 h after PH (Fig. 3D,E), depending on the mitochondrial activity in hepatocytes. The lipocalin‐2 (Lcn2) gene was highly expressed in clusters 1 and 2, whereas metallothionein (Mt) and orosomucoid (Orm) were overexpressed in cluster 2 compared to cluster 1. Metabolic pathway enrichment analysis was used for hepatocytes, and it was found that a higher inflammatory response after PH and the immune response contribute widely to liver regeneration (Fig. 3F).

In summary, hepatocytes in the quiescent phase after PH mainly maintain normal liver function, whereas those in the active phase experience proliferative and protective effects induced by the above pathways.

### Pseudotime analysis of hepatocytes

**Fig. 4:**
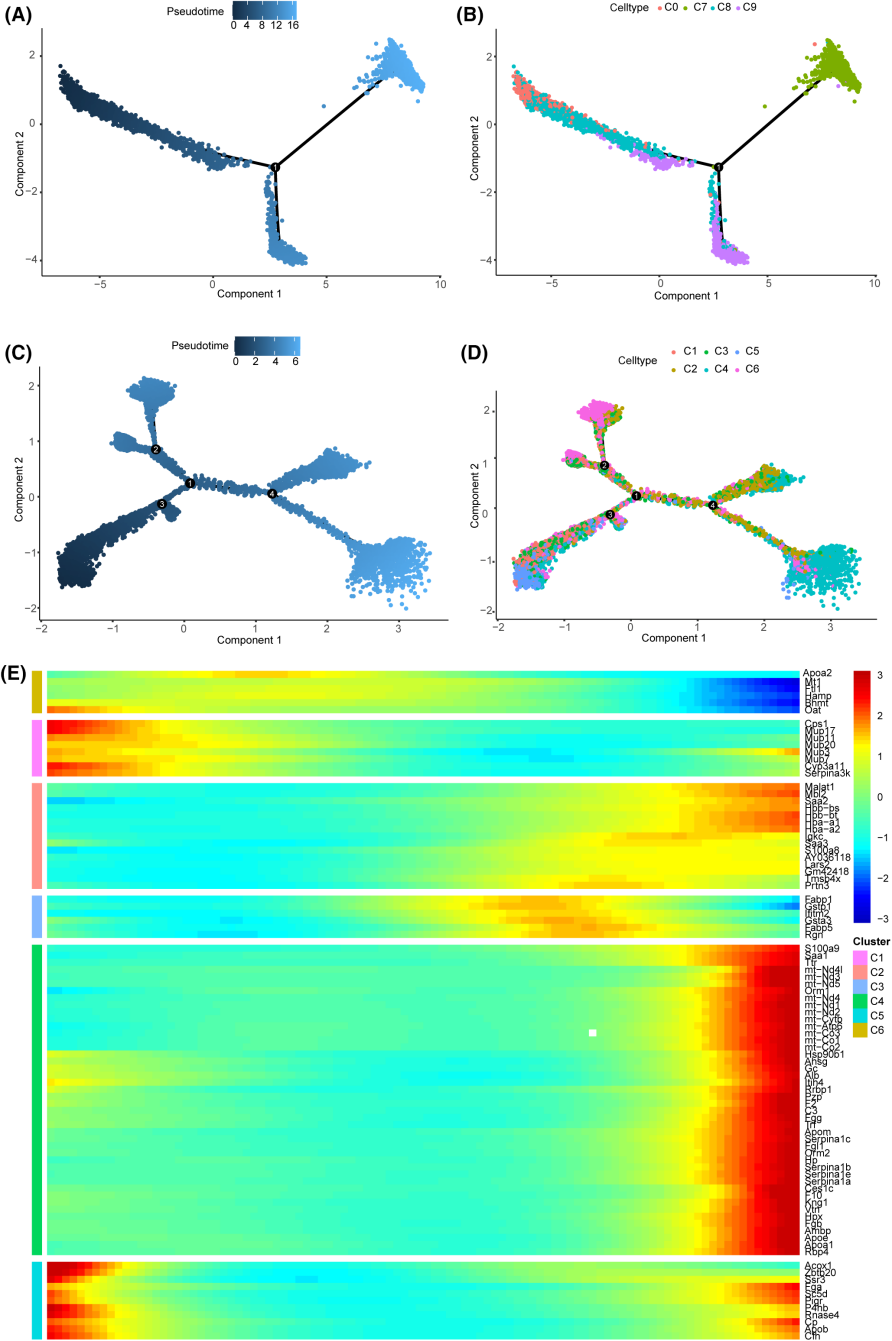
The dynamic change of hepatocytes after PH. (A) Pseudotime plot indicating cellular trajectories of hepatocytes from clusters 0, 7, 8 and 9 after PH. Single‐cell trajectories were constructed, and pseudotime values were calculated using monocle 2. The trajectories are colored according to the pseudotime. (B) Trajectory demonstrating the three distinct states of hepatocytes in clusters 0, 7, 8 and 9. (C) Pseudotime plot indicating cellular trajectories of hepatocytes from clusters 1–6 after PH. Single‐cell trajectories were constructed, and pseudotime values were calculated using monocle 2. The trajectories are colored according to pseudotime. (D) Trajectory demonstrating the three distinct states of hepatocytes in clusters 1–6. (E) Heat maps representing modules of genes in clusters 1–6 that co‐vary along the pseudotime at 0, 24 and 48 h after the PH renaturation phases.

We constructed discrete cell‐state trajectories from the initiation progression phase to the peak phase of regeneration of each state cell (PH 0 h → PH 24 h → PH 48 h). We found that PH 0 h, PH 24 h and PH 48 h quiescent cells resided on separate arms of the trajectory (Fig. 4A,B), similar to the previous study in Fig. 2B. Cluster 0 accounted for the major part 0 h after PH. Clusters 8 and 9 were transitioning cells and cluster 7 was highly expressed at 24 and 48 h after PH.

Using the same approach, proliferative cells were analyzed, as shown in Fig. 4C, and were separated into six branches of the trajectory. Cluster 5 was located in the PH 24 h pseudotime arm. Clusters 4 and 6 were located in the PH 48 h pseudotime arm (Fig. 4D). In addition, to identify the continuous changes within differential genes along the pseudotime, we performed a dynamic analysis of the top 10 genes that varied as a function of progress through the pseudotime. Along the 0–48 h pseudotime trajectory, the expression of Hamp, Oat and Apoa2 declined, and S100a8, S100a9, mt, Rnase4 and Mup3 simultaneously increased. Interestingly, genes for Gstp1, Eabp1, Eabp5, Ifitm2, Gata3 and Rgn first increased at 24 h and then decreased at 48 h (Fig. 4E).

Our analysis successfully captures the cellular plasticity of the hepatocytes. This result highlights that not all hepatocytes undergo reprogramming after PH. The dramatic change in hepatocytes after PH balances mature liver function and liver regeneration.

### Hepatocytes performed a diversified proliferative function in three zones

**Fig. 5:**
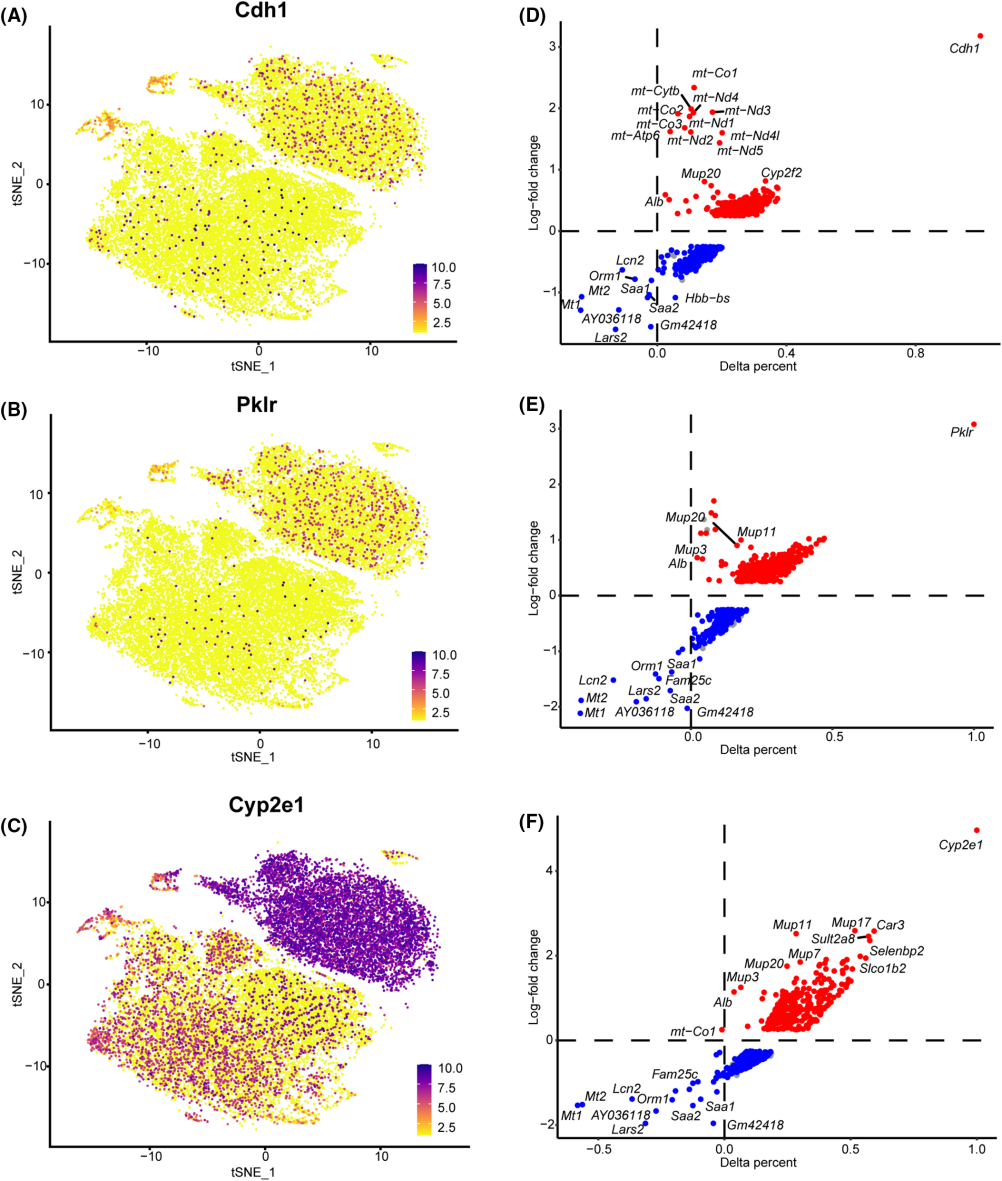
Hepatocytes performed a diversified proliferative function in three zones. (A) Zone 1 hepatocytes were identified based on the zone 1 marker gene Cdh1, using the t‐SNE algorithm. (B) Zone 2 hepatocytes were identified based on the zone 2 marker gene Pklr, using the t‐SNE algorithm. (C) Zone 3 hepatocytes were identified based on the zone 3 marker gene Cyp2e1, using the t‐SNE algorithm. (D) The expression patterns of key genes in zone 1 are shown as volcano plots. (E) The expression patterns of key genes in zone 2 are shown as volcano plots. (F) The expression patterns of key genes in zone 3 are shown as volcano plots.

Based on the different microenvironments, including oxygen levels, nutrients and secreted factors, the liver lobule can be roughly divided into three distinct zones: the periportal zone (zone 1), the pericentral zone (zone 3), and the mid‐lobular zone (zone 2) between zone 1 and zone 3 [27]. A recent study has reported that a subpopulation of hepatocytes located in zone 2 preferentially contributed to new hepatocytes during liver homeostasis and regeneration [28, 29]. Therefore, we aimed to explore the heterogeneity of hepatocytes in the three zones after PH. We identified zone 1 (Cdh1), zone 2 (Pklr) and zone 3 (Cyp2e1) based on their specific marker genes as previously reported (Fig. 5A–C). As shown in Fig. 5D, the mt‐Atp6, mt‐Co1, mt‐Co2 and mt‐Co3 genes were up‐regulated in zone 1, which indicates that mitochondrial metabolism is boosted in zone 1 to afford liver regeneration. However, hepatocytes in zones 2 and 3 showed high expression of Mup3, 7, 11, 17 and 20, suggesting that the hepatocytes may function in normal liver metabolism and rarely contribute to the proliferation in zones 2 and 3 (Fig. 5E,F). The hepatocytes in zone 1 were more active than in zones 2 and 3, whereas hepatocytes in zones 2 and 3 balanced the normal liver function from 0 to 48 h after PH.

### Neutrophils increased after PH

**Fig. 6:**
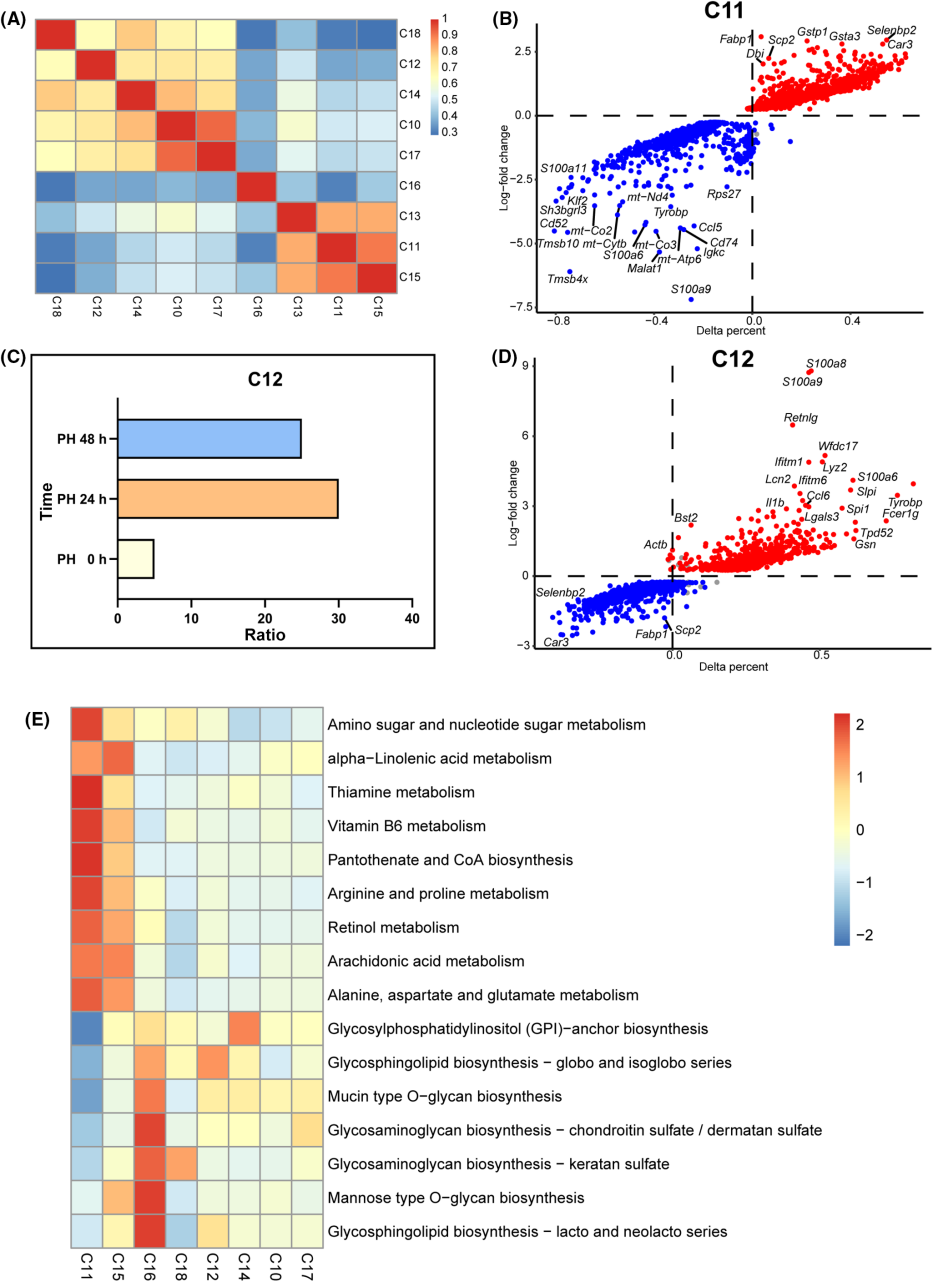
Neutrophils increased after PH. (A) Heat map of the relative expression of the top genes enriched in non‐parenchymal cells. The intensities and directions of the correlations are indicated on the right side of the heatmap. (B) Expression patterns of the critical genes in cluster 11 are shown in volcano plots. (C) Proportions of neutrophils in 0, 24 and 48 h after PH using the sc‐RNAseq database. (D) Expression patterns of the critical genes in cluster 12 are shown in volcano plots. (E) Heatmap showing KEGG pathway analysis results of marker genes for clusters 10–18.

Non‐parenchymal cells, particularly immune cells, have been reported to contribute to liver regeneration. We analyzed the relationship in each cluster of NPCs (Fig. 6A). Cluster 11 was identified as macrophages (Eabp5, Gstp1). Cluster 11 also highly expressed diazepam binding inhibitor during liver regeneration (Figs 1A and 6B), which has been reported to stimulate mitochondrial fatty acid oxidation and regulate the cell cycle [30]. Cluster 12 was identified as neutrophil (sp100a, sp100b) and was dramatically increased from 0 to 48 h after PH as shown in Fig. 6C. Cluster 12 also highly expressed Lcn2, interleukin (IL)1b, Ccl6 and galectin 3, which were inflammatory‐related genes (Fig. 6D). Figure 6E shows that NPCs were enriched in amino sugar and nucleotide sugar metabolism, indicating that NPCs also participate in liver regeneration by regulating metabolic pathways. The dramatic changes of neutrophils at 24 and 48 h after PH raised our attention with expect to exploring the function of neutrophils in liver regeneration.

### Depletion of neutrophils damaged liver regeneration

**Fig. 7:**
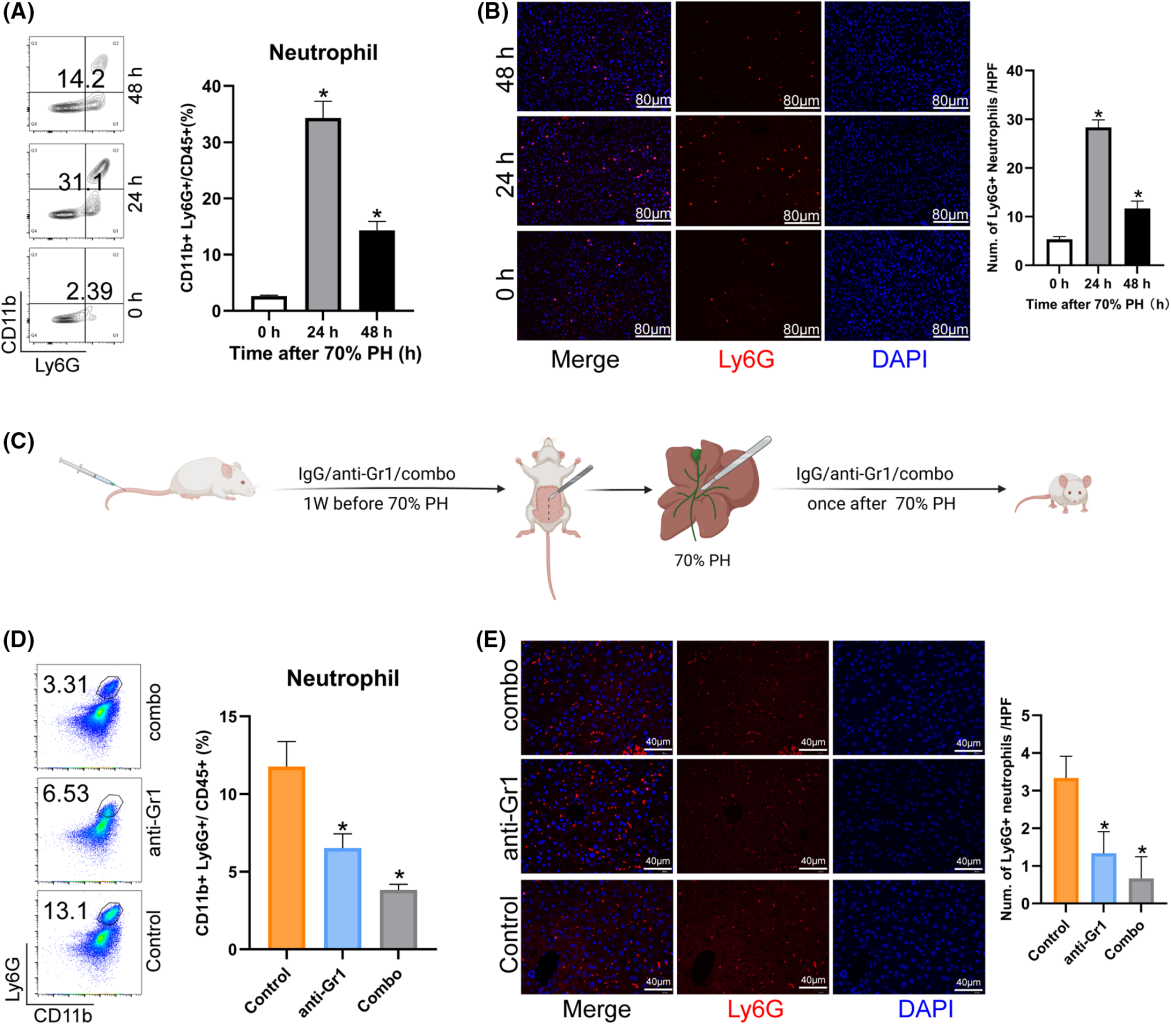
Neutrophils increased after PH. (A) Flow cytometry showed the percentage of neutrophils (Ly6G^+^CD11b^+^) among total immune cells (CD45^+^) after PH (*n* = 6, *P < 0.05). (B) Immunofluorescence staining of Ly6G^+^ neutrophils time after PH (*n* = 3, *P < 0.05). (C) Schematic representation of neutrophil depletion by PH (control: rat IgG2a isotype; anti‐Gr1: anti‐Gri antibody; combo: anti‐Ly6G antibody with anti‐rat IgG2a). (D) Flow cytometry showed the percentage of neutrophils (Ly6G^+^CD11b^+^) in total immune cells in different groups (CD45^+^) (*n* = 6, *P < 0.05). (E) Immunofluorescence staining of Ly6G^+^ neutrophils in different groups (*n* = 3, *P < 0.05).

First, we verified the variation tendency of neutrophils using flow cytometry and immunofluorescence, and it had the same results as our analysis (Fig. 7A,B). Next, we investigated the function of neutrophils in liver regeneration. We used neutrophil‐specific depletion antibody to delete the mouse liver neutrophils and performed a mouse PH model to explore the role of neutrophils (Fig. 7C–E).

**Fig. 8:**
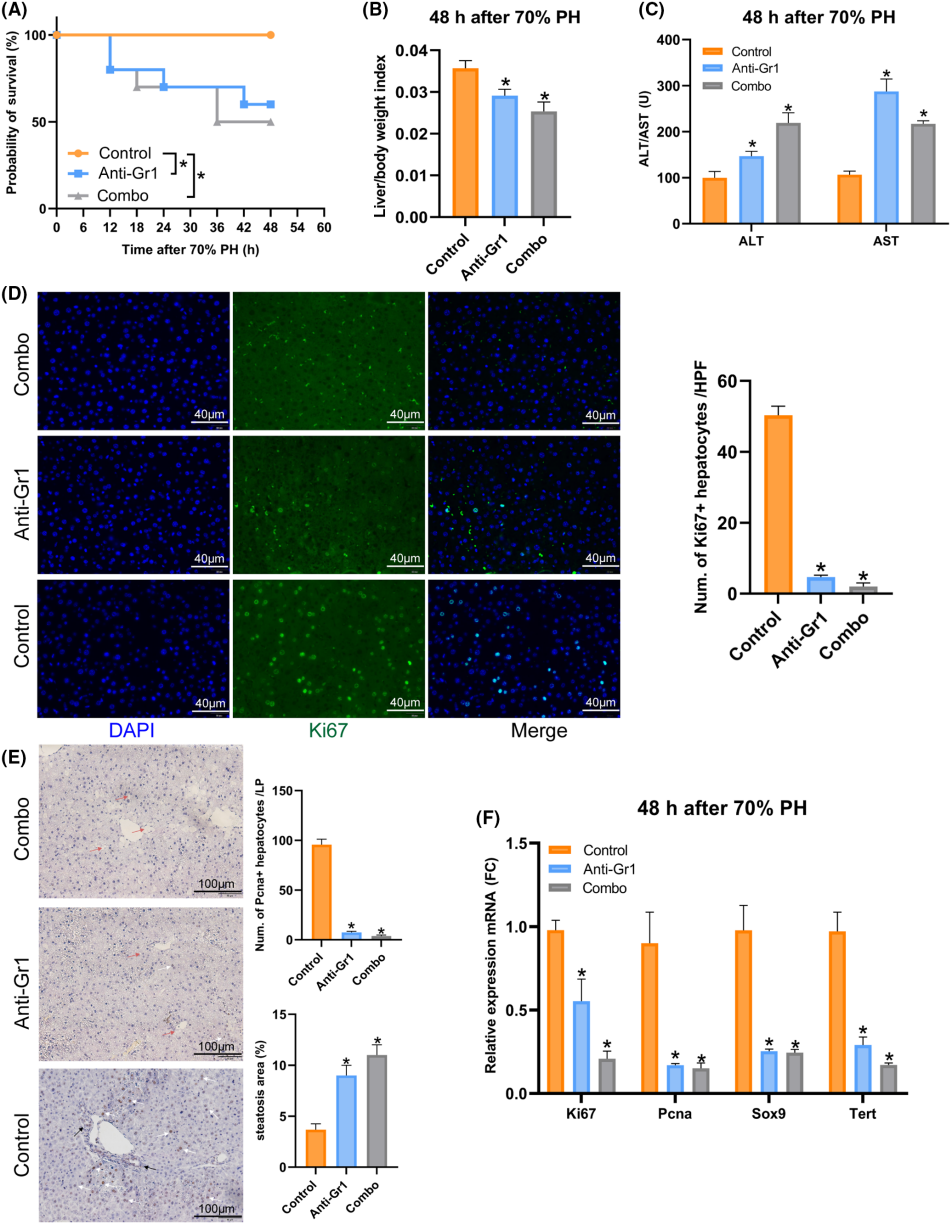
Depletion of neutrophils damaged liver regeneration. (A) Kaplan–Meier analysis was used to determine the survival of mice 48 h after PH (control: rat IgG2a isotype; anti‐Gr1: anti‐Gri antibody; combo: anti‐Ly6G antibody with anti‐rat IgG2a, *n* = 10, *P < 0.05). (B) The liver‐to‐body weight ratio after 48 h. Liver to body weight ratio = liver weight/body weight (g·g^−1^) (*n* = 5, *P < 0.05). (C) Serum levels of transaminase‐ALT and AST are shown (*n* = 5, *P < 0.05). (D) Immunofluorescence staining of marker of proliferation Ki‐67 (Ki67) and the number of Ki67^+^hepatocytes are shown (*n* = 3, *P < 0.05). (E) Immunohistochemical staining of proliferating cell nuclear antigen (Pcna) and the number of Pcna^+^ hepatocytes and steatosis areas are shown (white arrow: Pcna^+^ hepatocytes; black arrow: Pcna^+^ hepatocytes around the bile duct in zone 1; red arrow: lipid droplet in the liver, *n* = 3, *P < 0.05). (F) The mRNA expression levels of Ki67, Pcna, Sox9 and Tert were examined using qRT‐PCR. Genes expression was normalized to the GAPDH mRNA levels in each sample (*n* = 3, *P < 0.05).

As shown in Fig. 8A–C, the survival rate, the liver‐to‐body weight ratio, and the serum liver transaminase–ALT and AST levels were severely damaged after PH when neutrophils were depleted. The number of proliferating hepatocytes (Ki67^+^ and Pcna^+^ hepatocytes) decreased after depletion (Fig. 8D–F). In addition, the steatosis area increased, indicating the metabolic dysfunction in the absence of neutrophils after PH (Fig. 8E). The mRNA level also showed that proliferative rate and stem cell marker levels decreased when neutrophils depleted (Fig. 8F). All of these results suggest that neutrophils play an indispensable role in liver regeneration and maintenance of normal function.

### Crosstalk between neutrophils and macrophages promotes liver regeneration

**Fig. 9:**
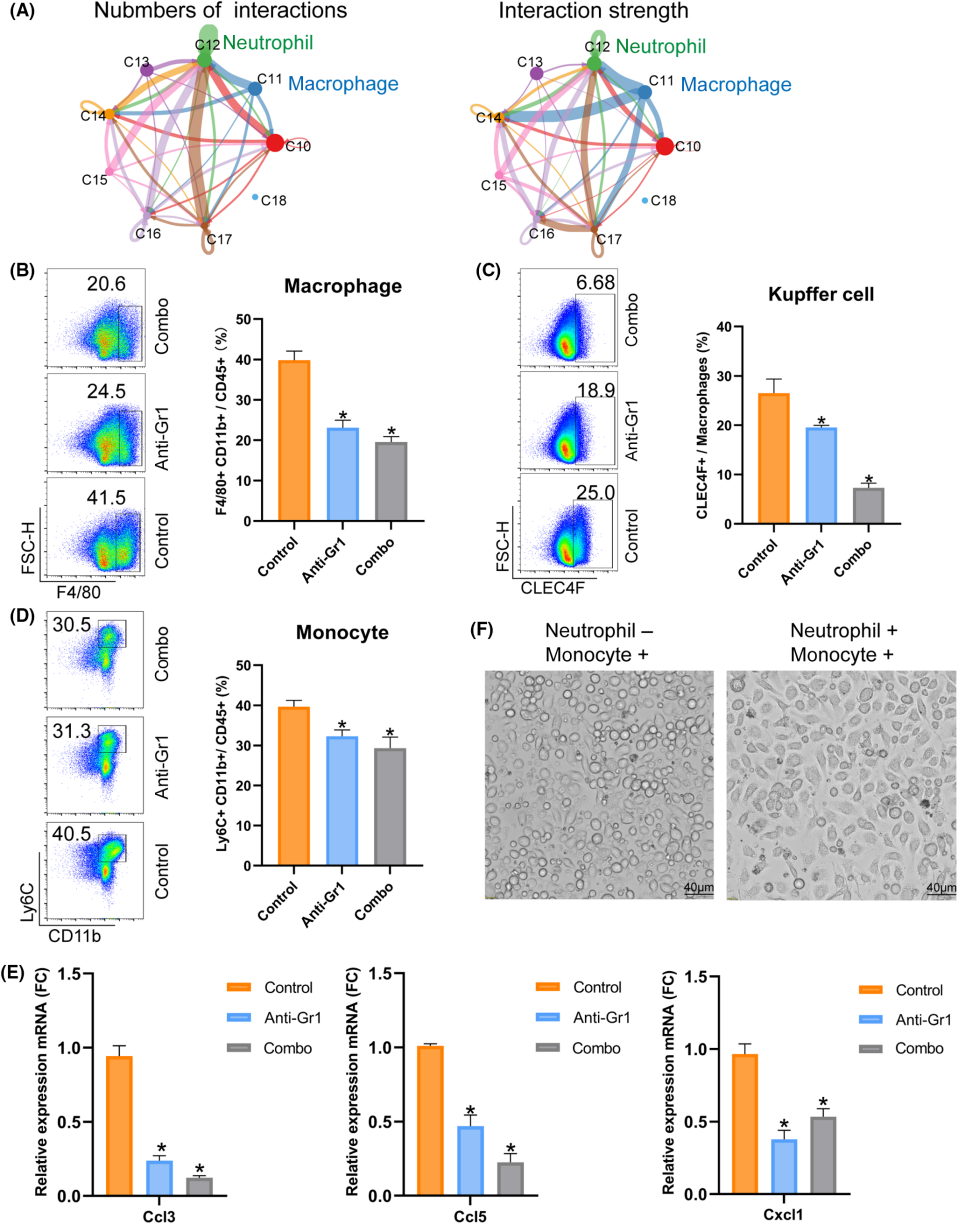
The interaction between neutrophils and macrophages enhances liver regeneration. (A) Network diagrams showing cell–cell interactions indicated by arrows (edges) pointing in the source‐to‐target direction. The thickness indicates the sum of the weighted paths between populations, and the color of the arrows corresponds to the source. Network diagrams for clusters 10–18 are shown. (B) Flow cytometry was used to determine the percentage of macrophages (F4/80^+^CD11b^+^) in total immune cells (CD45^+^) in the different groups (*n* = 6, *P < 0.05). (C) Flow cytometry revealed the percentage of Kupffer cells (CLEC4F^+^) in macrophages in the different groups (F4/80^+^CD11b^+^CD45^+^) (*n* = 6, *P < 0.05). (D) Flow cytometry revealed the percentage of monocytes (Ly6C^+^CD11b^+^) among the total immune cells in the different groups (CD45+) (*n* = 6, *P < 0.05). (E) The mRNA expression levels of Ccl3, Ccl5 and Cxcl1 were examined using qPCR analysis (the genes were normalized to GAPDH mRNA levels in each sample. Ccl3, C‐C motif chemokine ligand 3; Ccl5, C‐C motif chemokine ligand 5; Cxcl1, C‐X‐C motif chemokine ligand 1; *n* = 3, *P < 0.05). (F) Macrophages cultured without neutrophils and co‐culture of neutrophils and macrophages (*n* = 3, *P < 0.05).

Finally, we used CellChat analysis to explore the interactions between NPCs (Fig. 9A). The number and strength in cluster 12 (S100a8, S100a9) with respect to other clusters were the strongest, indicating that neutrophils had the most substantial relationship with other cells. The interactions between clusters 11 and 12 were the strongest, indicating that neutrophils had stronger crosstalk with macrophages.

Interestingly, the percentages of macrophages, monocytes and Kupffer cells all decreased after the depletion of neutrophils (Fig. 9B–D). This can be explained by the depletion of Ly6C^+^ monocytes when anti‐Gr1 (Ly6C/Ly6G) antibody was used. In total, monocytes also decreased after the depletion of neutrophils, whereas monocytes were beneficial for liver regeneration via transfer into necrotic foci to phagocytose dead cell debris [31]. The number of macrophages also decreased after PH because the neutrophils may secrete C‐C motif Ccl3, Ccl5 and C‐X‐C motif chemokine ligand (Cxcl)1 to recruit macrophages (Fig. 9E). The co‐culture of neutrophils and macrophages also confirmed the neutrophils stimulated monocytes to differentiate into macrophages (Fig. 9F). These results suggest that the crosstalk between neutrophils and macrophages promotes liver regeneration after PH.

### WGCNA identified regeneration‐related modules

Because most HCC patients present at a high age and experience some liver diseases [6], such as hyperlipidemia and even non‐alcoholic fatty liver disease, the metabolic environment also influences liver regeneration [7]. Here, we analyze the dynamic changes of the liver with different clinical traits, such as aging, high fatty diet (HFD) and regeneration following the PH mouse model to explore the different proliferative capabilities of these populations.

**Fig. 10:**
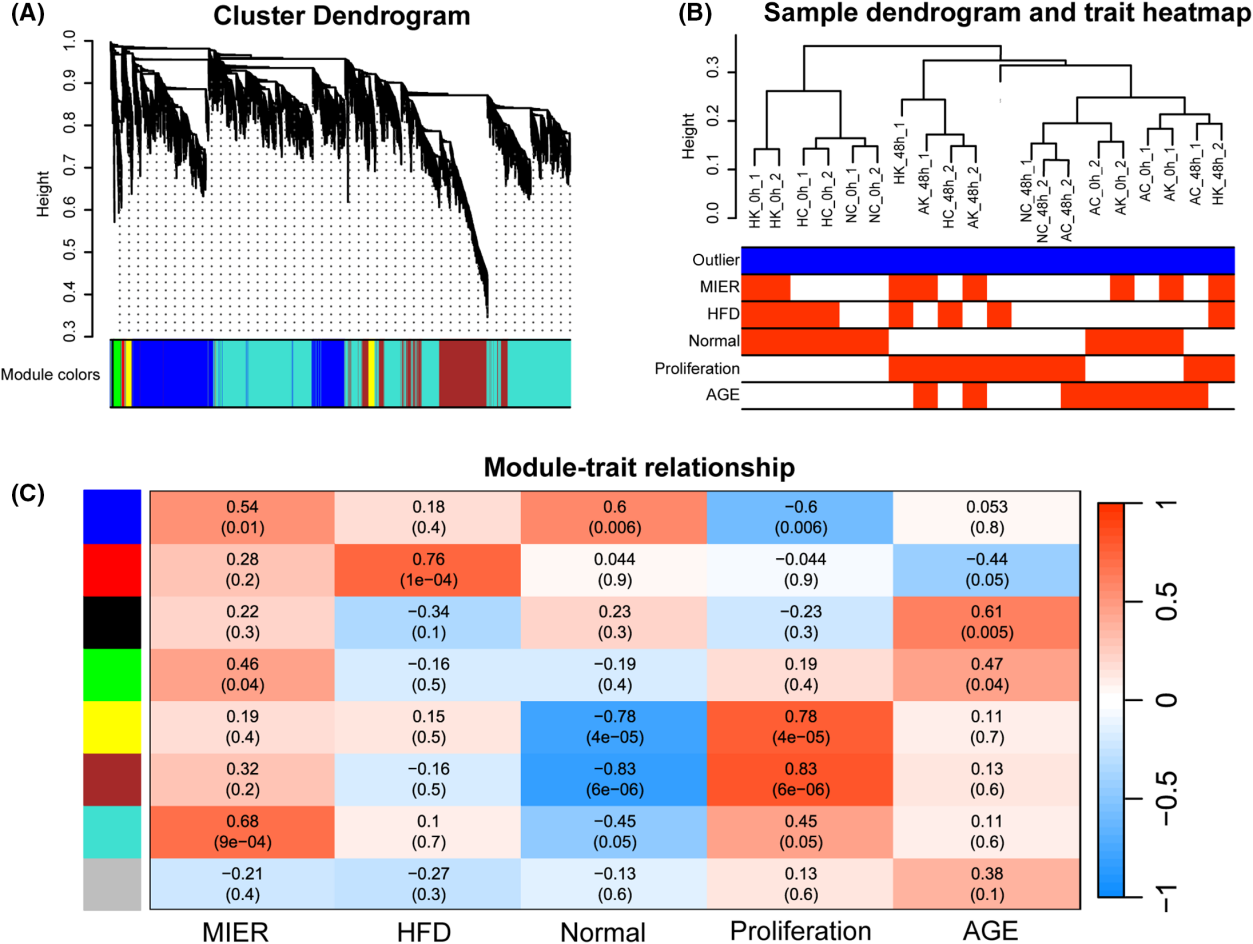
Construction of weighted gene co‐expression network identification of modules. (A) Gene expression pattern‐based DEGs clustering and module screening. The top is the gene dendrogram, and the bottom is the gene modules with different colors. In total, seven modules were identified in this study (the gray module represents genes that do not belong to any modules). (B) Sample dendrogram and trait heatmap of each clinical trait. (C) Relationship between consensus module eigengenes and clinical traits. The module is displayed on the left side of each cell. The numbers in the table indicate the corresponding module eigengenes and traits correlations, with the *P*‐values printed below the correlations in parentheses. The table is color‐coded according to the color legend. The intensities and directions of the correlations are indicated on the right side of the heatmap.

**Fig. 11:**
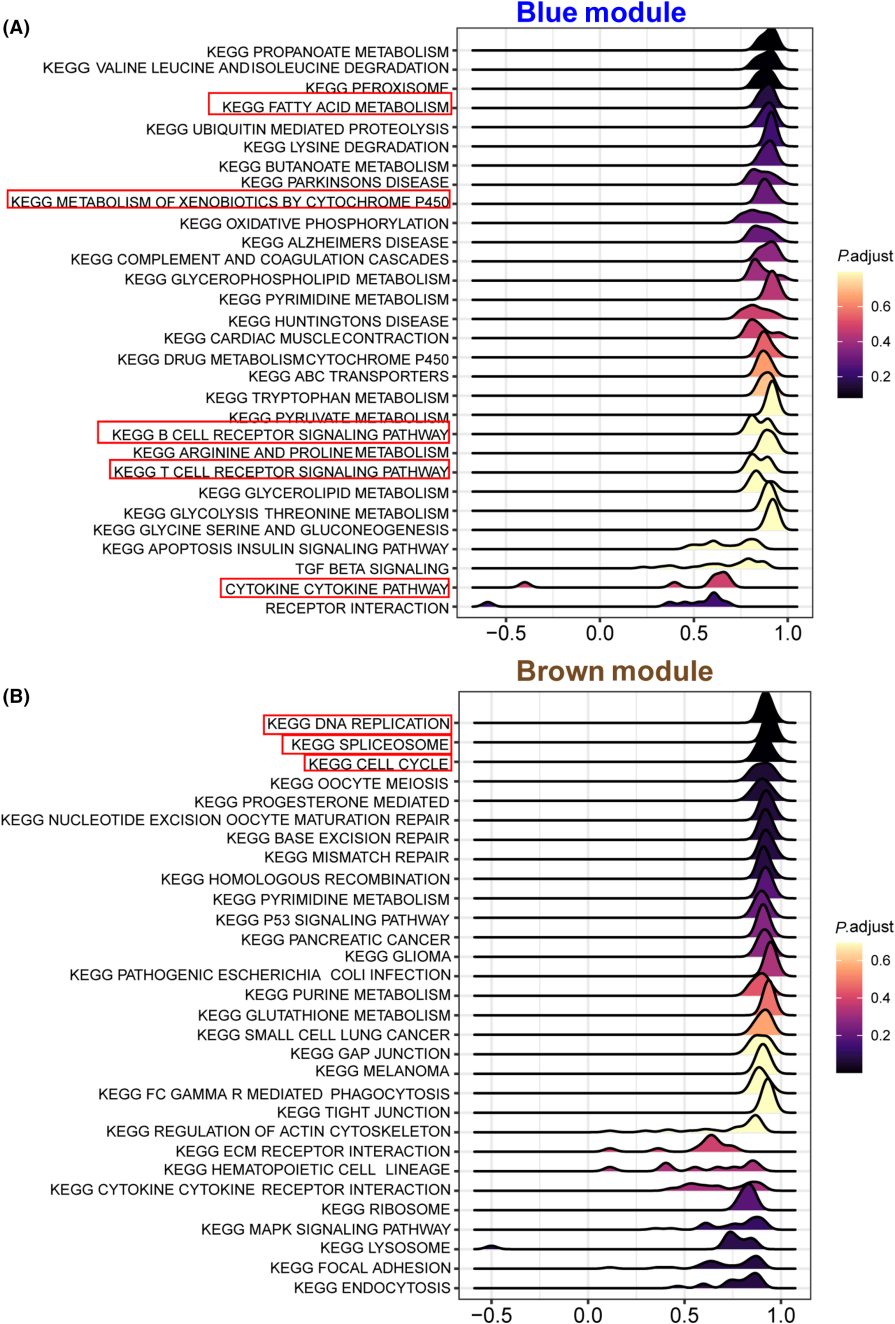
The metabolic pathways related to the clinical traits. (A, B) KEGG pathway enrichment analysis of the (A) blue and (B) brown module genes. Pathway names are shown on the left.

We constructed a WGCNA to determine the relationship between clinical traits and liver regeneration (Fig. 10A). The module‐trait heatmap represents the correlations between the module genes and clinical characteristics (Fig. 10B). We identified seven modules shown as different colors in Fig. 10C (the gray module represents genes do not belong to any modules). The yellow and brown modules were the most significantly correlated with liver regeneration. Next, we performed KEGG pathway analysis of different modules to determine the pathway involved in each characteristic. Blue module was characterized as normal liver. In Fig. 11A, several greatly enhanced pathways in the blue module, including fatty acid metabolism and drug metabolism cytochrome P450, are needed to balance the normal liver function after PH. Together with the T cell, B cell receptor signaling cytokine‐cytokine pathways, the most relevant modules related to activating liver regeneration are essential for controlling hepatocyte proliferation and paracrine cell interactions [32]. Brown module was most closely related to proliferation. As shown in Fig. 11B, DNA replication, spliceosome and cell cycle pathways were significantly enriched to adapt to the increasing need for cell regeneration after PH in the brown module.

## Discussion

In the present study, we have tracked the cellular transitions and heterogeneities of the liver using a scRNA‐seq database in a mouse PH model. We found that the dramatic change of hepatocytes after PH balanced mature liver function and liver regeneration. Importantly, we found that immune cells play an important role during liver regeneration, and the interaction between neutrophils and macrophages enhanced liver regeneration after PH.

The regeneration capacity of hepatocytes is most important for liver regeneration both in homeostasis and injury. Our study found that quiescent cells contribute to normal liver function and the proliferative cells contribute to liver regeneration, and these two types of populated hepatocytes balanced the liver regeneration after PH. Cluster 2 in 48 h was more active than cluster 1 in 24 h, according to the high expression of the gene Mt1 and Mt2 in cluster 2. Mt is critical in protecting the liver from oxidative stress‐induced liver injury by activating NF‐κB target genes [33]. Cluster 2 also highly expressed Orm compared to cluster 1. Orm is one of the major acute‐phase proteins in humans and is mainly synthesized in the liver, also playing an important role in liver regeneration [34]. We found that hepatocytes in zone 1 were more active than zone 2 and 3, whereas hepatocytes in zone 2 and 3 mainly contributed to normal metabolism during 0–48 h after PH. However, recent research has confirmed that liver regeneration following PH is enabled by the proliferation of hepatocytes throughout the liver, rather than by a particular zone [35, 36]. It is possible that such a discrepancy is a result of the different time points after PH that we selected. Our findings indicate that, overall, hepatocytes in zone 1 were more active than those in zones 2 and 3 from 0 to 48 h after PH. In addition, we only gathered the highly expressed genes to evaluate the activation of the specific population of hepatocytes, which focused on cell division, whereas other research focuses on the differentiation of stem cells or other cells [36, 37, 38].

Importantly, our research confirmed the existing results that NPCs, especially immune cells, function as necessary in liver regeneration [8]. Hepatocyte reprogramming is closely associated with the dynamic injured‐induced immune responses that play crucial roles in the initial time of liver regeneration. Our study focused on the dynamic change of neutrophil count, and we verified the indispensable role of neutrophils after liver resection (Fig. 7). In addition, we used CellChat analysis and revealed that neutrophils have stronger crosstalk with macrophages. The depletion of neutrophils impaired the recruitment of macrophages and Kupffer cells (KCs) and further decreased liver regeneration, and the co‐culture of neutrophils and monocytes accelerated the monocytes to differentiate into the macrophages to help liver regeneration after PH (Figs 8 and 9). It was also previously reported that neutrophils and macrophages interact with each other and accelerate liver regeneration [39]. CD11/CD18 in neutrophils bind with intercellular adhesion molecule‐1 in KCs and induce the release of interleukin‐6 and tunor necrosis factor‐α to accelerate hepatocytes into the cell cycle [40]. Activated KCs directly influence the recruitment of neutrophils through the secretion of Cxcl2, which binds to Cxcr2 in neutrophils [41]. KCs can also indirectly promote neutrophil recruitment by stimulating other cell types, such as hepatic stellate and endothelial cells [42]. Interestingly, our results showed that neutrophils secreting chemokines Cxcl1, Ccl3 and Ccl5 to recruit macrophages are stimulants during PH, and the interaction between neutrophils and macrophages is important for liver regeneration. Our study also found Lcn2 upregulated at 24 and 48 h after PH, whereas the ability of neutrophils to perform chemotaxis for transmigration depended on a neutrophil secondary Lcn2. LCN are members of a family of evolutionarily conserved genes and participate in non‐alcoholic steatohepatitis by promoting neutrophil‐macrophage crosstalk via the induction of CXCR2 [39] and influence macrophage polarization to promote liver regeneration [43].

The liver balances cell proliferation and metabolism to prevent liver function disorders or failure after PH [26]. Hepatocytes have a flexible metabolic machinery that can adapt dynamically to changes during tissue regeneration. Following PH, vital liver functions must be preserved except for cell division, such as maintaining blood glucose homeostasis, balancing the degradation of hepatic glycogen stores and gluconeogenesis [44]. Simultaneously, a distinct population of metabolically hyperactive cells appears to compensate for the temporary deficits in liver function [45]. Our study focused on liver metabolism and its dynamic regulation during liver regeneration, and we found that fatty acid metabolism was strongly overexpressed 24 and 48 h after PH to offer extra energy needed for liver regeneration.

However, the present study also had some limitations. There was no in‐depth research into the mechanism of neutrophils and liver regeneration. In addition, WGCNA did not gather the most significant pathways in the HFD module and age modules, which cannot provide any advice for the age or HFD populations when they go through PH.

## Conclusions

Given that most patients with HCC have underlying liver disease, it is meaningful to promote liver regeneration after liver transplantation or resection. Our study unraveled the regenerative capacity of hepatocytes at diverse temporal points after PH, unveiling the contributions of three distinct zones in the liver regeneration process. Our study also explored the crosstalk in NPCs and the dynamic metabolism changes in the liver, also indicating a potential target for liver regeneration treatment.

## Conflicts of interest

The authors declare that they have no conflicts of interest.

### Peer review

The peer review history for this article is available at https://www.webofscience.com/api/gateway/wos/peer‐review/10.1002/2211‐5463.13803.

## Author contributions

WS and XX designed and conceived the study. CYY and YYJ conducted most of the experiments and prepared the manuscript. LJJ and CH provided technical support for the animal experiments. CYY, NX and WXD performed the RNA‐sequencing data analysis. SZX revised the manuscript. YYJ, LJJ, CH, WXD and SZX reviewed the manuscript. All of the authors read and approved the final version of the manuscript submitted for publication.

## Data Availability

ScRNA‐seq data of mouse PH samples were downloaded from the GEO database (GEO: GSE151309); bulk RNA‐seq data of mouse PH samples were downloaded from the GEO database (GEO: GSE212628). Any other datasets generated in the present study are available from the corresponding author upon reasonable request.
